# rDNA magnification is a unique feature of germline stem cells

**DOI:** 10.1073/pnas.2314440120

**Published:** 2023-11-15

**Authors:** Jonathan O. Nelson, Tomohiro Kumon, Yukiko M. Yamashita

**Affiliations:** ^a^Whitehead Institute for Biomedical Research, Cambridge, MA 02142; ^b^HHMI, Chevy Chase, MD 20815; ^c^Department of Biology, Massachusetts Institute of Technology, Cambridge, MA 02142

**Keywords:** ribosomal DNA, rDNA copy number maintenance, germline immortality, Drosophila germline

## Abstract

Ribosomal DNA (rDNA) encodes ribosomal RNA and exists as tandem repeats of hundreds of copies in the eukaryotic genome to meet the high demand of ribosome biogenesis. Tandemly repeated DNA elements are inherently unstable; thus, mechanisms must exist to maintain rDNA copy number (CN), in particular in the germline that continues through generations. A phenomenon called rDNA magnification was discovered over 50 y ago in Drosophila as a process that recovers the rDNA CN on chromosomes that harbor minimal CN. Our recent studies indicated that rDNA magnification is the mechanism to maintain rDNA CN under physiological conditions to counteract spontaneous CN loss that occurs during aging. Our previous studies that explored the mechanism of rDNA magnification implied that asymmetric division of germline stem cells (GSCs) may be particularly suited to achieve rDNA magnification. However, it remains elusive whether GSCs are the unique cell type that undergoes rDNA magnification or differentiating germ cells are also capable of magnification. In this study, we provide empirical evidence that suggests that rDNA magnification operates uniquely in GSCs, but not in differentiating germ cells. We further provide computer simulation that suggests that rDNA magnification is only achievable through asymmetric GSC divisions. We propose that despite known plasticity and transcriptomic similarity between GSCs and differentiating germ cells, GSCs’ unique ability to divide asymmetrically serves a critical role of maintaining rDNA CN through generations, supporting germline immortality.

## Significance

Tandem repetitiveness of ribosomal DNA (rDNA) makes it unstable, posing the challenge in maintenance particularly in the “immortal” germline, which forms continuous lineage of cells and organisms. Two competing models [unequal sister chromatid exchange (USCE) model and extrachromosomal rDNA circle reintegration model] have been proposed as a mechanism underlying rDNA magnification, a process identified over 50 y ago to recover rDNA copy number (CN). The present study provides empirical evidence and computer simulation that support the USCE model and provide explanations to observations that were previously deemed inconsistent with the USCE model. A revised model proposes that asymmetric stem cell division of germline stem cells is the critical cell biological aspect that enables USCE to effectively increase rDNA CN.

**Fig. 1.**
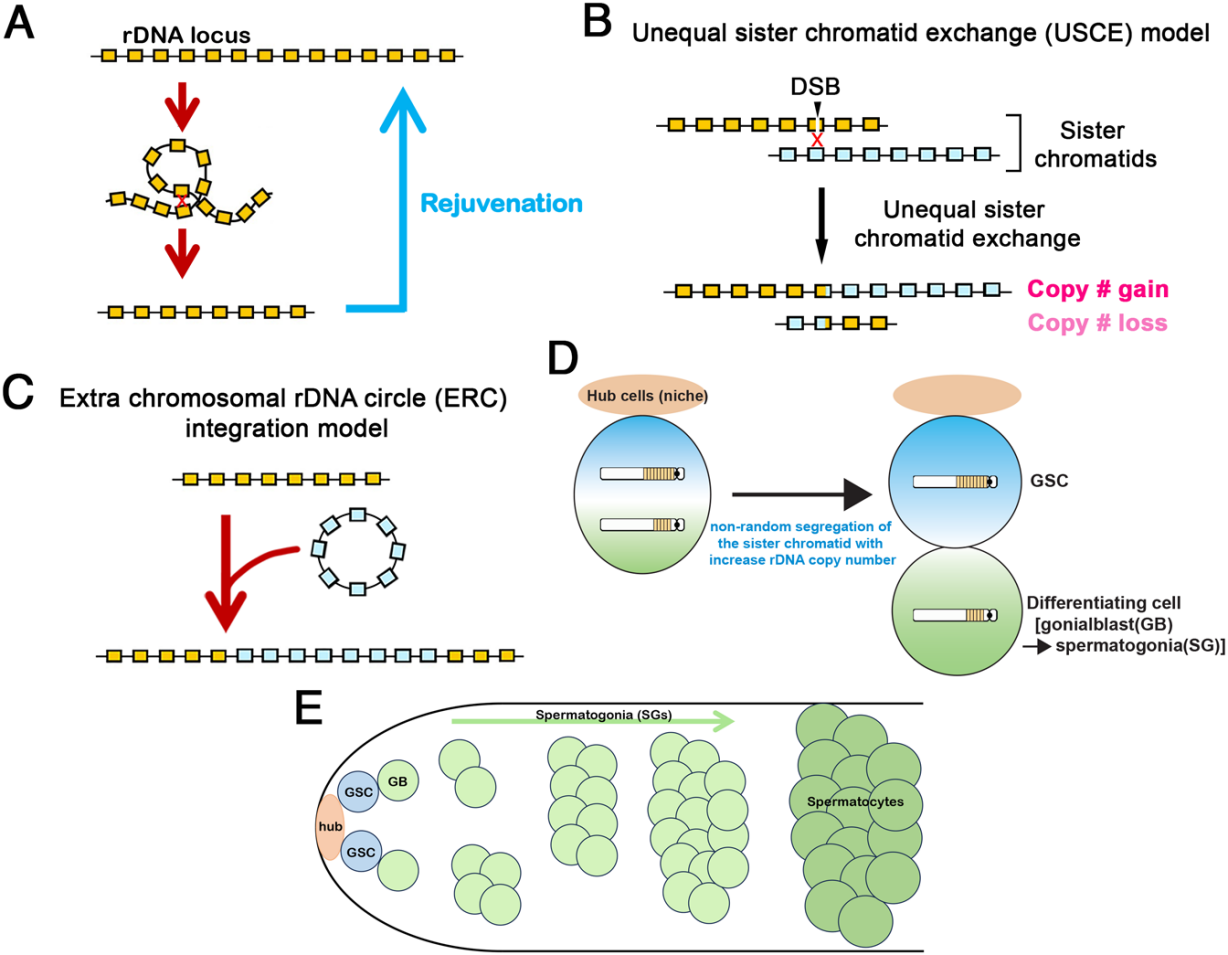
Model of rDNA magnification. (*A*) The rDNA locus is inherently unstable, requiring a mechanism to recover the CN for germline immortality. (*B*) USCE as a model of rDNA CN expansion (rDNA magnification). (*C*) Reintegration of extrachromosomal rDNA circle as a model of rDNA magnification. (*D*) Nonrandom sister chromatid segregation following USCE increases rDNA CN in GSCs (a model from ref. 3). (*E*) Schematic of germ cell differentiation in the Drosophila testis. GSCs reside at the apical tip of the testis, where they attach to somatic hub cells, which functions as the major component of the stem cell niche. GSCs divide asymmetrically to produce gonialblasts (GBs), which undergo mitotic proliferation to produce a cluster of interconnected spermatogonia (SGs). Upon 4 mitotic divisions, SGs enter meiotic prophase as spermatocytes.

Ribosomal DNA (rDNA) loci harbor hundreds of tandemly repeated ribosomal RNA (rRNA) genes to meet the high demand of ribosome biogenesis, with rRNA synthesis constituting ~80% of total cellular transcription in the cell (1). Tandemly repeated DNA elements, including rDNA, are inherently unstable due to sporadic intrachromatid recombination, which removes intervening copies while generating circularized DNA (Fig. 1*A*). In yeast, rDNA instability is an established cause of replicative senescence, and the mechanisms exist to recover rDNA copy number (CN), involving sister chromatid recombination (2).

The germline of multicellular organisms is the only cell type that transmits the genome to the next generation, constituting the immortal lineage of the cells and organisms. Accordingly, germlines must have mechanisms to counteract spontaneous rDNA CN loss. *Drosophila melanogaster* has long served as an excellent system to monitor rDNA CN changes, particularly because it has only two rDNA loci (one on each sex chromosome, X and Y), while most other model multicellular organisms have several rDNA loci (4, 5), making it difficult to track the rDNA CN changes on each chromosome. Furthermore, collections of chromosomes with partial or complete deletions of the rDNA loci have been isolated in *D. melanogaster*, facilitating experimental manipulation of rDNA CN (4). Usefully, flies that have a minimal rDNA CN exhibit various developmental defects, collectively called a “bobbed phenotype”, which allows visual identification of low rDNA CN (including thin bristles and abnormal cuticles) (4). Using these chromosomes with reduced rDNA CN (“bobbed chromosomes”), a phenomenon called “rDNA magnification” was discovered over 50 y ago as a process to increase rDNA CN in the male germline, causing some progeny of bobbed males to regain wild-type characteristics (6, 7). rDNA magnification also likely serves in wild-type animals to maintain rDNA CN through generations to offset spontaneous CN loss that occurs during the aging of individual animals (8).

Earlier studies led to two major models as the potential mechanism of rDNA magnification. One model proposed that unequal sister chromatid exchange (USCE) at rDNA loci allows one chromatid to “steal” copies from its sister, producing sister chromatids that have reciprocally gained and lost rDNA copies (Fig. 1*B*, USCE model) (9). The other model proposed that extrachromosomal rDNA circles (ERC) produced by intrachromatid recombination at rDNA may undergo amplification and reintegrate into the rDNA loci, thereby recovering rDNA CN (Fig. 1*C*, ERC reintegration model) (10). Both models have suffered contradictory experimental observations. The fact that the recovered repeats were always duplicates of large local stretches of rDNA copies (instead of assemblies of rDNA copies from multiple locations) disfavored the ERC reintegration model, while favoring the USCE model (11). However, the USCE model also suffered observations that are inconsistent with the predictions. Since USCE-dependent rDNA CN increase relies on “stealing” of copies from the sister chromatid, this model predicts that the CN gain is at maximum twofold (one chromatid stealing all copies from its sister), yet greater than twofold CN gains have been observed during rDNA magnification (12). With these observations inconsistent with the existing models, the underlying mechanism of rDNA magnification remained enigmatic to this day.

Recently, we reported mechanisms that contribute to rDNA magnification. We observed that under the magnifying condition, dividing GSCs (germline stem cells) exhibited asymmetry in rDNA CN, with higher rDNA CN being preferentially inherited by GSCs than its differentiating daughter, leading us to propose that GSCs gained rDNA CN via USCE (Fig. 1*D*) (3). GSCs are attached to the somatic hub cells, which function as the stem cell niche, and undergo repeated asymmetric divisions, generating one self-renewed GSC and one differentiating cell called GB (gonialblast) (Fig. 1 *D* and *E*) (13, 14). GBs undergo four rounds of symmetric differentiating divisions as SG (spermatogonia), producing 16 meiotic spermatocytes (SC), each capable of creating 4 mature sperm (Fig. 1*E*) (14, 15). It has been proposed that rDNA magnification predominantly occurs during these mitotic divisions to allow for much larger effects than USCE could achieve during meiosis, although there is evidence that USCE in meiotic germ cells also contributes to a small amount of rDNA magnification (12, 16). This model, however, was developed prior to the detailed description of GSC asymmetric divisions (17, 18), and the ability to directly assess USCE in mitotic germ cells has remained limited. Accordingly, the relative contribution of asymmetric GSC divisions and symmetric differentiating divisions to rDNA magnification has remained largely unexplored.

We also reported that the retrotransposon R2, which specifically inserts into the 28S rDNA sequence (19), is required for rDNA magnification and transgenerational maintenance of rDNA CN (20). The ability of R2 to generate DNA breaks at rDNA loci is likely utilized as the triggering event to catalyze USCE at rDNA loci (20, 21). However, the activation of retrotransposons poses a major threat to genome stability; thus, they are normally silenced within the germline (22–25). It remains unclear how this threat is minimized when R2 is expressed to induce rDNA magnification or how R2 expression may differently impact GSCs and differentiating germ cells.

In this study, by using Drosophila genetics, cytology, and computer simulation, we investigated how the process of rDNA magnification is integrated in the biology of asymmetric GSC divisions and symmetrically dividing differentiating cells (GBs/SGs). We provide evidence that rDNA magnification is a unique feature of GSC biology. In contrast, ectopic activation of R2 in differentiating germ cells is harmful, leading to marked reduction in the number of differentiating germ cells. Collectively, we propose that GSCs’ unique characteristic to divide asymmetrically is critical for rDNA CN maintenance and thus germline immortality.

## Results

### DNA Double-Strand Breaks (DSBs) Are Limited to GSCs.

**Fig. 2.**
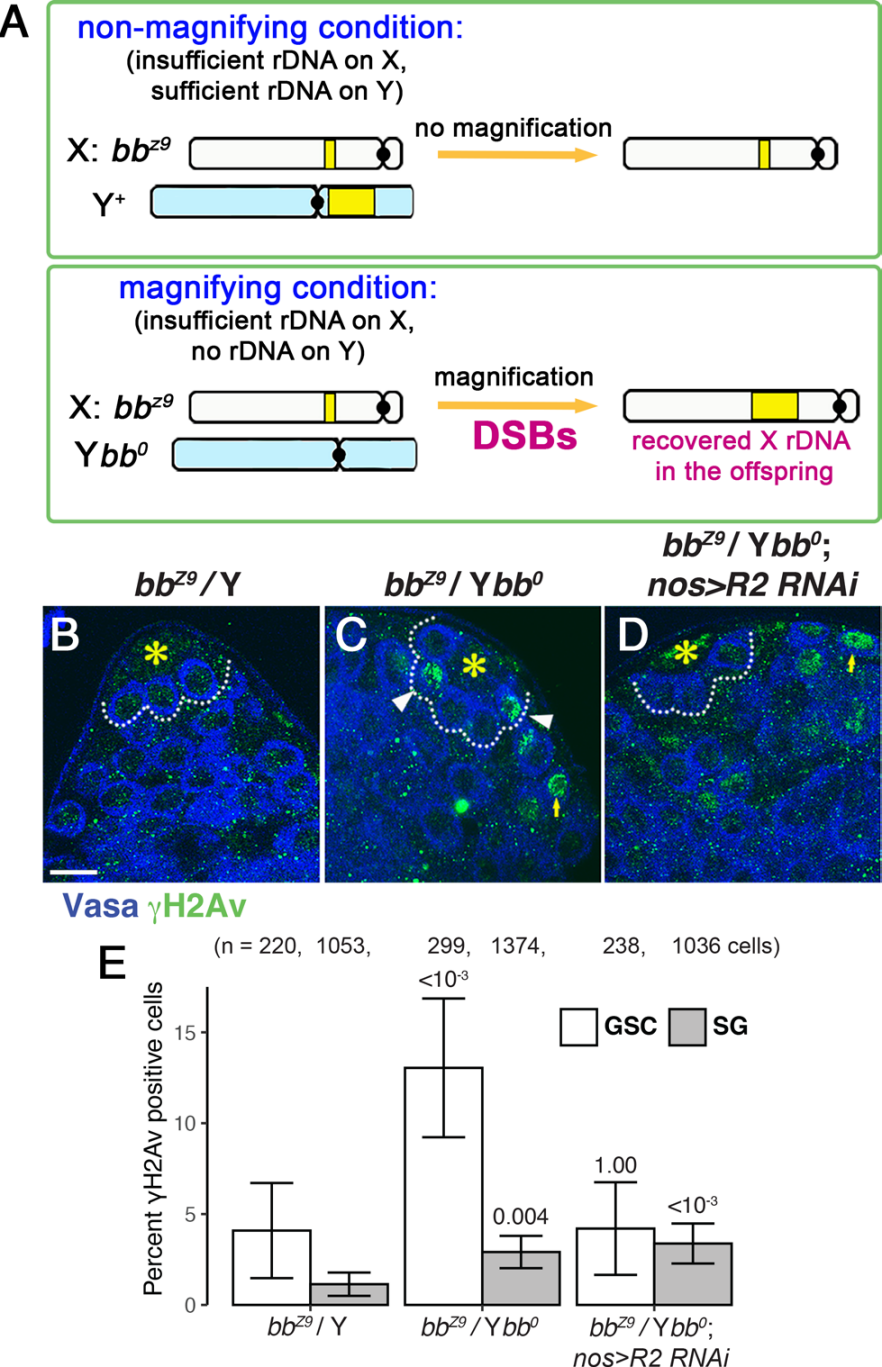
R2-dependent double-strand DNA breaks under low rDNA CN occur primarily in GSCs. (*A*) rDNA magnification occurs only when animals have insufficient rDNA CN (*bb^z9^*/Y*bb^0^*; magnifying condition), whereas animals with sufficient rDNA CN (*bb^z9^*/Y^+^ or X^+^/Y^+^, nonmagnifying conditions). (*B*–*D*) Representative images of the apical tip of the testes from control (*bb^z9^*/Y^+^) (*B*), *bb^z9^*/Y*bb^0^* (*C*), and *bb^z9^/Ybb^0^ nos-gal4 > R2 RNAi* (*D*) testes, stained for γH2Av (marker for DSBs, green) and Vasa (germ cells, blue). Asterisks (*) mark the hub. The dotted line indicates GSCs. White arrowheads indicate DSB-positive GSCs, and yellow arrows indicate DSB-positive SGs. (Bar: 10 µm.) (*E*) The frequency of γH2Av-positive GSCs vs. SGs in indicated genotypes. DSBs are most prominently elevated in GSCs under magnifying conditions (*bb^z9^*/Y*bb^0^*), which is repressed by RNAi-mediated knockdown of R2. *P* values displayed compared to the control condition in the same cell type using chi-squared test. Error bars = 95% CI.

Although rDNA magnification likely occurs in wild-type animals to support transgenerational maintenance of rDNA CN (8), rDNA magnification has been typically assayed using animals that harbor minimal rDNA CN, allowing for visual assay using bobbed cuticle phenotype caused by insufficient rDNA CN (6, 7). In particular, males harboring an X chromosome with critically low rDNA CN [e.g., *bb^Z9^* used in this study, see *Methods* (20)] are used to observe rDNA magnification, where the state of the *bb^Z9^* X chromosome (whether it recovered rDNA CN or not) can be assayed in the offspring (Fig. 2*A*). When *bb^Z9^* is combined with a Y chromosome completely lacking rDNA (*Ybb^0^*), the total low rDNA CN induces magnification, leading to CN recovery on *bb^Z9^* locus (Fig. 2*A*). In contrast, when the *bb^z9^* X chromosome is paired with Y chromosome with normal rDNA CN, magnification does not occur because there is sufficient total rDNA CN due to the intact Y chromosome rDNA locus (Fig. 2*A*). The condition that triggers rDNA magnification (*bb^z9^*/Y*bb^0^*) is referred to as the “magnifying condition,” and the *bb^Z9^/Y* genotype that does not induce rDNA magnification is referred to as the “nonmagnifying condition” hereafter.

rDNA magnification is initiated by the formation of DSBs at the rDNA locus (26) (Fig. 1*B*). Note that while the initiation of USCE requires DSBs at the rDNA locus, other models such as reintegration of circular rDNA require DSBs as well (Fig. 1*C*). We have shown that GSCs increase DSBs under magnifying conditions (*bb^z9^*/Y*bb^0^*) (Fig. 2 *B*–*E*, white bars) (20). However, we found that under the same condition, the induction of DSBs is not as prominent in differentiating cells (SGs) (Fig. 2*E*, gray bars). This result suggests that SGs, unlike GSCs, are less responsive to low rDNA CN in inducing DSBs. Moreover, whereas RNAi-mediated knockdown of R2 eliminated DSB induction under magnifying conditions (20), R2 knockdown did not impact DSB frequency in SGs, implying that R2 may primarily operate in GSCs to induce DSBs under magnifying condition (Fig. 2*E*). These results suggest that DSB formation, the very first step of rDNA magnification, is primarily induced in GSCs but not in SGs in response to reduced rDNA CN. Accordingly, SGs are less likely to undergo rDNA magnification.

### R2 Expression Is Limited to GSCs.

Because the increased DSB formation in GSCs under magnifying conditions is dependent on R2, we reasoned that DSBs primarily being induced in GSCs but not SGs may be due to biased expression of R2 in GSCs compared to SGs under magnifying conditions (Fig. 2*E*).

**Fig. 3.**
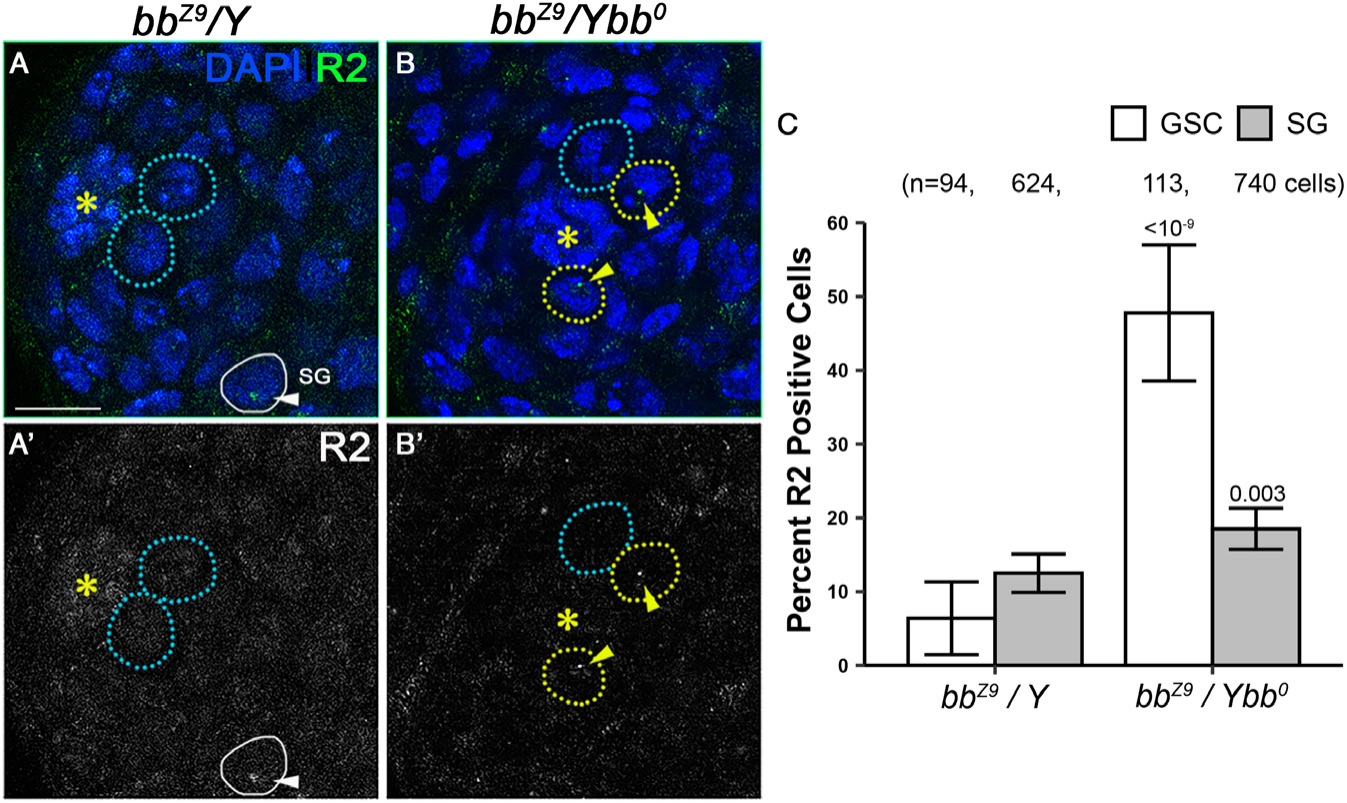
Induction of R2 expression under magnifying condition is enriched in GSCs. (*A* and *B*) Representative images of the testis apical tip from animals under nonmagnifying (*A*, *bb^z9^*/Y^+^) vs. magnifying (*B*, *bb^z9^*/Y*bb^0^*) conditions. DAPI: blue, R2 RNA: green. (*A’* and *B’*) R2 RNA in situ hybridization only channel in grayscale. Asterisks (*) indicate the hub. Broken blue circles indicate GSCs negative for R2 expression, broken yellow circles indicate GSCs positive for R2 expression (also indicated by arrowheads), and the white solid circle indicates SGs positive for R2 expression. (*C*) The frequency of R2-positive GSCs (white bars) and SGs (gray bars) under nonmagnifying (*bb^z9^*/Y^+^) vs. magnifying (*bb^z9^*/Y*bb^0^*) conditions. P values indicate comparison between genotypes among the same cell types using chi-squared test. Error bars = 95% CI.

We examined the expression of R2 by RNA in situ hybridization under nonmagnifying and magnifying conditions. RNA in situ hybridization allowed single-cell resolution, enabling us to determine in which cell type R2 is expressed (Fig. 3 *A* and *B*). R2 expression in GSCs was markedly increased under the magnifying condition compared to the nonmagnifying condition (20), whereas R2 expression increased only slightly in SGs (Fig. 3*C*), suggesting that induction of R2 due to low rDNA CN is mostly limited to GSCs.

### Computer Simulation Suggests that USCEs in GSCs, But Not SGs, Can Accomplish rDNA Magnification.

The fact that DSB formation and R2 expressions are more prominently induced in GSCs compared to SGs suggests that rDNA magnification may be a unique feature of GSCs. Moreover, we previously showed that R2’s requirement in rDNA magnification was limited to early germ cells (GSC to 2-cell stage SGs) because knockdown of R2 in >4-cell stage SGs did not have any impact on rDNA magnification (20), suggesting that rDNA magnification might occur predominantly in early germ cells, including GSCs.

To further assess this idea, we used computer simulation to evaluate the impact of USCE in GSCs vs. SGs on rDNA CN change. We considered two questions: 1) how does biased or unbiased inheritance of USCE products during asymmetric GSC divisions impact rDNA CN? and 2) how does USCE during symmetric SG divisions impact rDNA CN?

**Fig. 4.**
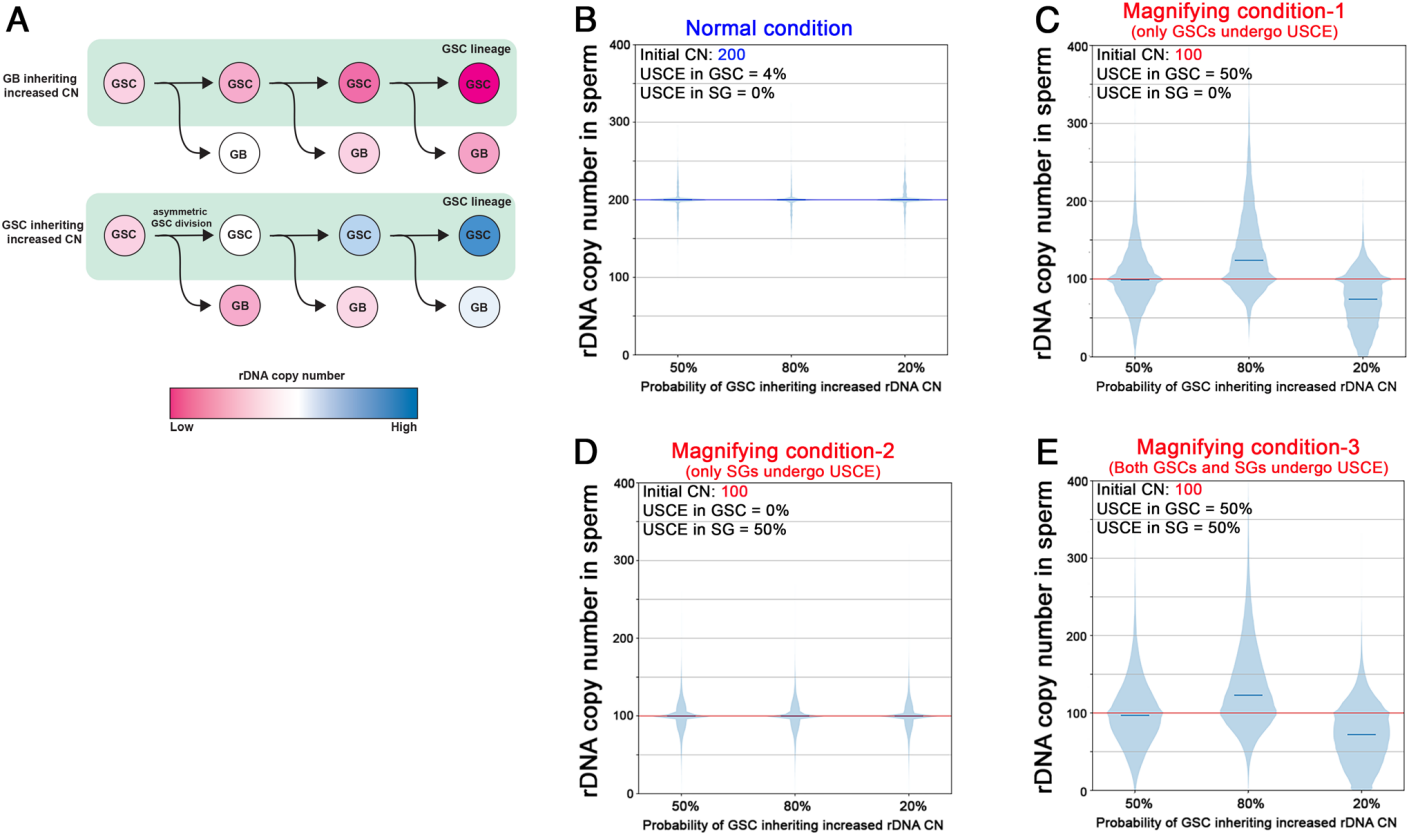
Simulation of rDNA CN changes based on the modes of rDNA CN inheritance. (*A*) Schematic model of rDNA CN changes over successive GSC divisions when GBs consistently inherit increased rDNA CN (*Top*), or GSCs inherit increased rDNA CN (*Bottom*). Note that differentiating cells (GBs) will receive successively decreasing rDNA CN when they inherit higher rDNA CNs than GSCs at each division. (*B*–*E*) Computer simulation of rDNA CN in sperm after 2 wk (or 28 GSC divisions). The initial rDNA CN was assumed to be 200 for nonmagnifying condition (*B*) and 100 for magnifying condition (*C*–*E*). The starting CN is shown in blue (*B*) or red (*C*–*E*). Other simulation parameters are also shown at the *Top Left* of each graph: the frequency of USCE in GSC and SG and the frequency of GSCs inheriting increased rDNA CN after UCSE (50%, 80%, and 20%). See the main text and *Methods* for details of parameter setting (some parameters are empirically determined based on previous studies).

The first question is prompted by empirical evidence suggesting that GSCs appear to inherit more rDNA CN than GB during magnification (3). However, these cytological observations do not provide direct evidence for USCE, and an argument can be made that GBs carry the genome that will be transmitted to the next generation; thus, it is better that GB inherits the sister chromatid that gained rDNA CN. However, in the scenario where GBs inherit sister chromatids that gained rDNA CN (Fig. 4 *A*, *Top*), GSCs would continue to lose rDNA CN, which results in all future GBs inheriting rDNA loci from GSCs that had lost the rDNA CN. Overall, in this scenario, it is expected that GBs’ rDNA CN (thus sperm’s rDNA CN) gradually decrease over time. In contrast, in the second scenario, where GSCs inherit sister chromatid that gained rDNA CN (Fig. 4 *A*, *Bottom*), GSCs gradually increase rDNA CN, and so do GBs: Although GBs inherit lower rDNA CN than GSC for each division, their rDNA CN will increase over successive GSC divisions because GBs’ DNA is synthesized using GSCs’ DNA, which continuously increase rDNA CN.

To validate these considerations more thoroughly, we conducted computer simulation to evaluate how the parameters (whether GSCs undergo USCE, whether GSCs inherit longer or shorter copies of rDNA, and whether SGs also undergo USCE) may affect rDNA CN. We modeled potential scenarios of USCE in mitotic germ cells, incorporating parameters drawn from empirical data (*Methods*). The initial rDNA CN was set either at 200 (for nonmagnifying condition) or 100 (for magnifying condition), although the pattern of rDNA CN increase or decrease over time is not expected to be impacted by initial CN. The frequency of GSC inheriting increased rDNA CN was set at 80%, 50% (random), or 20%. The frequency (80% or 20%) were chosen because we observed that the frequency of nonrandom sister chromatid segregation was around 80% (3, 27). Finally, the frequency of USCE was set at 4% (nonmagnifying condition) or 50% (magnifying condition). This is based on the frequency of DSBs observed in nonmagnifying conditions (4%, which is likely an overestimation because not all DSBs would lead to USCE) or the observed frequency of GSC anaphase with asymmetric rDNA amount under magnifying condition detected by DNA FISH (3).

Under “nonmagnifying” conditions, where the frequency of USCE is expected to be low (3), the computer simulation suggested that the overall rDNA CN in sperm did not change after 2 wk, with very little variation from the starting CN (Fig. 4*B*). Under magnifying conditions, when GSCs undergo USCE at 50% of divisions, and if GSCs inherited the improved rDNA CN at 80% of time, rDNA CN increased in majority of sperm (Fig. 4*C*, “80%”). The 80% inheritance of improved rDNA CN by GSCs also resulted in the appearance of a small number of sperm that lost rDNA CN. This is similar to the earlier observations that, during magnification, a single father can produce many offspring with expanded rDNA CN, while producing a small number of offspring with reduced rDNA CN (16). The small number of offspring with reduced rDNA CN likely represents the result of earlier GSC divisions, where GBs inherited lower- than-initial rDNA CN. It is of note that this condition (GSC inherit increased rDNA CN upon USCE) produced progeny that magnified more than twofold, i.e., starting from CN = 100, a considerable number of progeny reached CN > 200 (some reaching 400) (Fig. 4*C*), similar to the observation by de Cicco et al. (12). In contrast to the scenario where GSCs inherit improved rDNA CN, average rDNA CN in sperm did not increase if GSCs inherit the sister chromatid randomly with regard to the rDNA CN (Fig. 4*C*, “50%”). Although this condition resulted in a small number of progeny with improved rDNA CN, the degree of CN gain as well as the number of animals that gained rDNA CN was much less/fewer compared to when GSCs preferentially inherited the sister chromatid with recovered rDNA CN (Fig. 4*C*). Finally, if GSCs inherit the sister chromatid with decreased rDNA CN, rDNA CN in sperm decreased (Fig. 4*C*, “20%”), consistent with the reasoning explained in Fig. 4*A*. These models demonstrate that the biased inheritance of chromatids with improved rDNA CN by GSCs results in the most effective overall expansion of rDNA CN in sperm.

Although the low level of DSB formation and R2 expression in the GB/SG population (Figs. 2 and 3) indicate that these differentiating cells may not be heavily involved in rDNA magnification, we sought to examine how USCE in the GB/SG population may contribute to rDNA CN increase. To this end, we considered two scenarios: i) only GBs/SGs undergo USCE but GSCs do not, and ii) both GSCs and GBs/SGs undergo USCE. In these scenarios, the frequency of USCE in GB/SG is set at 50%, similar to the frequency of GSCs undergoing USCE during magnification (Fig. 4*C*), although this is clearly an overestimate, considering low DSB frequency and R2 expression in these cells (Figs. 2 and 3). When considering USCE in GBs/SGs only, varying inheritance patterns during GSC divisions (GSCs inherit improved rDNA CN at 80, 50, or 20% of chance) were still included in the simulation. The frequency of GSC undergoing such division was set to baseline nonmagnifying amounts (4%) for this scenario. Because GBs/SGs divide symmetrically, there is no need to consider the frequency of a cell inheriting improved rDNA CN (i.e., every USCE event produces one SG with expanded rDNA CN and one with reduced). Under these scenarios, average rDNA CN is not impacted, and rDNA CN increase only occurs in a small portion of offspring, with limited amounts of rDNA gained (Fig. 4*D*). Therefore, it is very unlikely that USCE in GBs/SGs alone can be the source of rDNA magnification.

While USCE in GBs/SGs alone does not appear to explain rDNA magnification, it is possible that USCE in these cells serves as a mechanism to enhance rDNA CN expansion in combination with USCE in GSCs. Therefore, we modeled the scenario where both GSCs and GBs/SGs undergo USCE (Fig. 4*E*). Similar to Fig. 4*D*, we modeled three distinct scenarios of GSCs inheriting improved rDNA CN at 80%, 50%, or 20% of the chance and set the frequency of USCE to 50%. Under all three GSC inheritance patterns, the results were similar to the scenario where only GSCs undergo USCE (Fig. 4*C*). In particular, in the scenario with GSCs inheriting improved rDNA CN at 80% of divisions, the condition that most efficiently expanded rDNA CN, there was no significant difference in mean rDNA CN between USCE in GSCs alone vs. both GSCs and GBs/SGs (equivalence test, see *Methods*), with the mean CN being 136.3 and 135.9, respectively. These results imply that USCE in GBs/SGs has little, if any, contribution to overall rDNA CN in sperm.

Taken together, our empirical evidence and computer simulation support a model of rDNA magnification, in which USCE in GSCs generates rDNA CN asymmetry, followed by biased inheritance of improved rDNA CN by GSCs. Moreover, GSCs are the major contributing source of rDNA CN expansion, whereas USCE in differentiating germ cells (GBs/SGs) has no major contribution to rDNA CN increase.

### Ectopic Expression of R2 in Differentiating Germ Cells leads to their loss.

The simulation suggested that USCE in GBs/SGs has minimal potential to facilitate rDNA magnification (Fig. 4 *D* and *E*). This implies that any DSBs produced in these cells during rDNA magnification are largely unproductive with regard to rDNA CN recovery. This follows that GBs/SGs may have no reasons to induce DBSs. Indeed, the frequency of DSBs is much lower in GBs/SGs (Fig. 2*E*). Not only are these DSBs in GBs/SGs unproductive, they may be harmful: Previously, we have shown that SGs are more sensitive to DNA damages than GSCs (28). SGs become more sensitive to DNA damages as their transit-amplifying divisions progress because SGs share their cytoplasm through incomplete cytokinesis, which allows sharing of cell death signal and lowers the threshold of damage required to trigger cell death (28). In this scenario, GSCs and GBs, both of which are not connected to other cells by cytoplasmic bridges, are the only germ cells that are not subjected to higher sensitivity to DNA damages. If this is the case, the DSBs needed to initiate USCE may trigger cell death in SGs that have higher sensitivity to DNA damages.

**Fig. 5.**
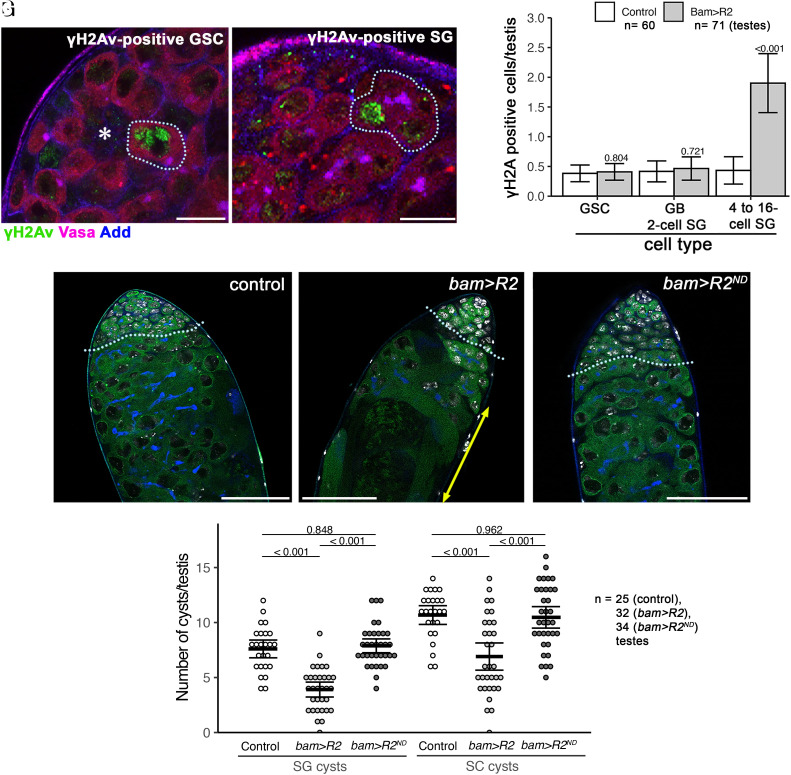
Ectopic expression of R2 in differentiating germ cells (>4-cell SG) reduces the number of differentiating germ cells. (*A* and *B*) Example of γH2Av-positive GSC (*A*) and SG (*B*) stained for γH2Av (green), Vasa (germ cells, magenta), and Adducin-like (blue). (Bars: 10 µm.) The asterisk (*) marks the hub (*A*), and the dotted line indicates γH2Av-positive GSC (*A*) or γH2Av-positive SG (*B*). (*C*) The frequency of γH2Av-positive cells/testis for the indicated stage of germ cells (GSCs/GBs and 2-cell SGs (*bam*-negative germ cells)/4- to 16-cell SGs (*bam*-positive germ cells) upon ectopic expression of R2 by *bam-gal4*. This result demonstrates that *bam*-positive (i.e., *bam-gal4*-expressing) germ cells specifically induce DSBs monitored by γH2Av. *P* values indicate a comparison between genotypes among the same cell types using Student’s *t* test. Error bars = 95% CI. n = numbers of testes scored from three biological replicates. (*D*–*F*) Representative images of the testis apical tip from control (*D*), *bam>R2* (*E*), *bam>R2^ND^* (nuclease-dead) at the age of 2 wk. The boundary between the SG stage and the SC stage is indicated by the dotted line. (Bar: 50 µm.) The yellow double-headed arrow in *E* indicates fully differentiated sperm (which moved toward the apical side due to loss of earlier germ cells, such as SGs and SCs). (*G*) Number of SG cysts and SC cysts per testis upon expression of R2 or nuclease-dead R2 (R2^ND^). *P* values displayed from Tukey’s HSD multiple comparison test after one-way ANOVA. ANOVA *P* values are > 0.00001 for both SG and SC analyses. Error bars = 95% CI. Data are from three biological replicates.

To test whether SG are sensitive to rDNA magnification-inducing DSBs, we expressed R2 in ≥4 cell SGs using *bam-gal4* driver (*bam > R2*) (29). We confirmed that *bam > R2* indeed induces DSBs (monitored by γH2Av staining) in ≥4 cell SGs, where *bam-gal4* is expected to be active (Fig. 5 *A*–*C* and *SI Appendix*, Table S1).

Upon expression of R2, the gross anatomy of the testis appeared to be normal in newly eclosed males, compared to wild type. However, after 2 wk, we observed a marked decrease in the numbers of SG and postmitotic SC cysts (Fig. 5 *D*–*G*). The expression of nuclease-dead R2, which is not expected to have the ability to generate DNA breaks (20), did not cause the reduction in the number of germline cysts (Fig. 5 *F* and *G*), suggesting that R2’s ability to cause DNA breaks is toxic to highly interconnected SGs (i.e., *bam*-expressing SGs). Therefore, we propose that R2 is primarily up-regulated in GSCs under magnifying conditions to protect SGs from cell death, which have higher DNA damage sensitivity.

## Discussion

Because rDNA repeats are essential but unstable genetic loci, it is critical to maintain their CN for the germline lineage to continue. This is akin to the need of telomere maintenance for germline immortality (30, 31). rDNA and telomere likely constitute two major genomic elements that require active maintenance in the germline.

rDNA magnification was discovered over 50 y ago as a phenomenon that recovers rDNA CN on “*bobbed (bb)*” chromosomes, which have lower-than-threshold rDNA CN (6, 7). The extensive studies on rDNA magnification using *bb* chromosome have generated the foundational framework of rDNA CN maintenance (6, 9, 10, 16, 21, 32–36). However, two major competing models (USCE model and ERC reintegration model) both suffered inconsistent observations, which have not been fully resolved to date.

These earlier studies predated the knowledge of cell biological characteristics of germline cells. The present study, aided by the knowledge of germ cell behaviors (e.g., asymmetric stem cell divisions, symmetric SG divisions), supports the USCE model and explains the observations that were originally deemed inconsistent with USCE as the source of rDNA magnification. First, the emergence of offspring that recovered rDNA CN more than twofold was considered to be inconsistent with the USCE model unless multiple USCE events occurred in successive germline mitoses (12). The present study demonstrates that repeated asymmetric GSC divisions can achieve such rDNA CN recovery within a single generation and is most effective if expanded rDNA loci are biasedly inherited by GSCs. Second, magnification is accompanied with a much lower frequency of rDNA reductions than rDNA expansion, which was considered to be inconsistent with the USCE model, as each USCE reaction would result in reciprocal CN gain and loss (leading to the same number of magnified and worsened offspring) (9, 16). It was speculated that the germ cells bearing reduced rDNA loci are selected against, leading to a higher frequency of magnified offspring than worsened offspring. Our simulation suggests that asymmetric GSC divisions may explain why there are many more magnified offspring than worsened offspring: Segregation of expanded rDNA loci to the GSC continues to increase rDNA CN and continues to produce progeny using the DNA template with recovered rDNA CN. Thus, lower-than-initial rDNA CN is produced only during the early processes of magnification, and the differentiating germ cells that inherited lower-than-initial rDNA CN only contribute to a limited number of progeny (Fig. 4*A*).

Our study reveals an interesting “division of labor” between GSCs and SGs, where GSCs are specialized for rDNA magnification through USCE and SGs are specialized in the maintenance of genome integrity with their higher sensitivity to DNA damage. This division of labor may provide an answer as to why GSCs undergo consistent asymmetric divisions. The asymmetric fate of GSC division (producing one GSC and one GB) was initially interpreted as simply a mechanism to maintain tissue homeostasis by generating one stem cell and one differentiating cell (37). However, it was later discovered that GBs and SGs often undergo dedifferentiation to replace lost GSCs (38–41), revealing cell fate plasticity between GSCs, GBs and SGs, and casting doubt on the notion that asymmetric division is necessary to sustain germline homeostasis. Indeed, many stem cell populations, including rodents’ spermatogonial stem cells, are sustained by symmetrically dividing stem cell populations (42–47). Recent single-cell RNA-seq studies also corroborated that there are only small transcriptional differences between GSCs, GBs and SGs (48, 49), consistent with plasticity between these cells. If these early germ cells’ fates are indeed so plastic, it leaves the question as to why the system has evolved many elaborate cell biological mechanisms to ensure asymmetric GSC divisions (38, 50–55). The present study indicates that the difference between GSCs and GBs/SGs may not be distinct “cell identity” reflected in transcriptomic differences, but rather asymmetry in rDNA CN. Moreover, because rDNA magnification is not triggered unless rDNA CN become lower than threshold, the transcriptome of GSCs and GBs/SGs may not become distinct unless cells have low rDNA CN. Indeed, R2 upregulation under magnifying conditions is mostly limited to GSCs, meaning there are critical differences in R2 regulation between GSCs and GB/SG, despite their close transcriptional identity. How R2 expression is regulated by the host’s transcriptional program to achieve this precise cell type activation only under magnifying conditions remains elusive.

In summary, the present work establishes GSCs as a unique cell population capable of achieving USCE-mediated rDNA magnification, thereby maintaining germline immortality. Our study provides an insight into how characteristic properties of these specific cell types contribute to the mechanisms that maintain vulnerable rDNA loci (and perhaps other tandemly repeated DNA), a common challenge most eukaryotes face.

## Materials and Methods

### Fly Husbandry.

Drosophila lines used in this study were *y w bb^Z9^* (20), *y^1^ eq^1^/Df(YS)bb^–^* (DGRC #101260), *y w; nos-gal4* (56), *bam-gal4* (29), *UAS-R2 RNAi-1*, *UAS-R2*, and *UAS-NucleaseDead R2*) (20). All animals were reared at 25º on standard Bloomington medium without propionic acid.

### Immunofluorescence Staining.

Testis Immunofluorescence staining was performed as previously described (20). In short, testes were dissected in 1× PBS, fixed in 4% formaldehyde in 1× PBS for 30 min, and then briefly washed two times in 1× PBS containing 0.1% Triton-X (PBS-T), then washing in PBS-T for 30 min. Following these washes, samples were incubated overnight at 4 °C with primary antibody (1:20 rat anti-vasa; Developmental Studies Hybridoma Bank (DSHB); developed by A. Spradling, 1:200 mouse anti-Fasciclin III; DSHB; developed by C. Goodman, and 1:200 rabbit anti-γ-H2AvD pS137; Rockland) in 3% bovine serum albumin (BSA) in PBS-T. Samples were washed in PBS-T three consecutive times for 20 min, followed by overnight incubation at 4 °C with secondary antibody in 3% BSA in PBS-T. After secondary antibody incubation, samples were again washed in PBS-T for three 20-min periods and then mounted in VECTASHIELD with DAPI (Vector Labs). The following primary antibodies were used: rat anti-vasa (1:20; DSHB; developed by A. Spradling), mouse anti-Fasciclin III (1:200; DSHB; developed by C. Goodman), and rabbit anti-γ-H2AvD pS137 (1:200; Rockland). Samples were imaged using a Leica Stellaris 8 confocal microscope with 63× oil-immersion objectives and processed using Fiji (ImageJ) software.

### RNA In Situ Hybridization.

RNA in situ hybridization samples were prepared as previously described (20). In short, dissected testes were fixed in 1× PBS with 4% formaldehyde for 30 min, briefly washed in 1× PBS, and permeabilized in 70% ethanol overnight at 4°. Samples were then briefly rinsed in 2× SSC with 10% formamide prior to overnight hybridization with 50 nM probes at 37°. Samples were washed twice in 2× SSC with 10% formamide for 30 min at 37° and mounted in VECTASHIELD with DAPI (Vector Labs). Samples were imaged using a Leica Stellaris 8 confocal microscope with 63× oil-immersion objectives and processed using Fiji (ImageJ) software. R2 Stellaris FISH probe set was designed and synthesized by Biosearch Technologies.

### Statistics.

For analyses that quantified percent γH2Av or R2 positive cells (Figs. 2 and 3), we pooled data across all samples within each condition to calculate the percentage of the entire dataset. Therefore, these analyses do not have individual data points. Accordingly, significance was determined by chi-squared test and error bars were generated using the CI for a Population Proportion formula. Comparisons of the number of γH2Av within each cell type (Fig. 5*C*) were determined by Student’s *t* test. For multisample comparison of germ cell cyst number (Fig. 5*G*), significant differences were first determined by one-way ANOVA, and *P* values of individual comparisons were determined by Tukey’s Honest Significant Difference (HSD) test.

### Computer Simulation.

For each stem cell lineage, the stem cell divides every 12 h for 2 wk (total 28 GSC divisions), and each stem cell division generates 16 sperm due to four mitotic divisions in SG/GB. For each round of stem cell division, USCE may occur in GSC or SG/GB at frequencies specified in each panel of Fig. 4. When USCE occurs, the number of rDNA CN changes follows the normal distribution with the mean and SD of the square root of the current CN based on the observation in ref. 3. The direction of CN change in GSC (gain or loss) is random (50%) or nonrandom (80% or 20%). The direction of CN change in SG/GB is random. The number of GSC is set to 10, and the number of flies is set to 100. Other parameters are specified in each panel of Fig. 4. Equivalence test to compare two groups (rDNA CN with USCE in GSCs alone ± 1 copy of rDNA vs. CN with USCE in both GSCs and GBs/SGs): Brunner-Munzel tests from both directions were used, which yielded *P* > 0.95 for both directions, supporting that the two groups might be different within 2 copies of rDNA, but they fall into this equivalence range. The distribution and the median of the rDNA CN in sperm are plotted. The custom code used to simulate the rDNA CN is available on GitHub (https://github.com/TomoKumon/).

## Supplementary Material

Dataset S01 (DOCX)

## Data Availability

All data are available in the manuscript or *SI Appendix*.
