# Supplementary material for: rDNA magnification is a unique feature of germline stem cells
Source: Proc Natl Acad Sci U S A. 2023 Nov 15;120(47):e2314440120. doi: 10.1073/pnas.2314440120 (PMC10666004; doi:10.1073/pnas.2314440120)
Supplement: Supplementary file 1 — Dataset S01 (DOCX) [file pnas.2314440120.sd01.docx]

| **Genotype** | **Cell Type** | Number of γH2Av positive cells | | | | | | | | | | | | | |
| --- | --- | --- | --- | --- | --- | --- | --- | --- | --- | --- | --- | --- | --- | --- | --- |
|  |  | **0** | **1** | **2** | **3** | **4** | **5** | **6** | **7** | **8** | **9** | **10** | **11** | **12** | **13** |
| Control | GSC | 39 | 19 | 2 | 0 | 0 | 0 | 0 | 0 | 0 | 0 | 0 | 0 | 0 | 0 |
|  | GB & 2-cell SG | 42 | 11 | 7 | 0 | 0 | 0 | 0 | 0 | 0 | 0 | 0 | 0 | 0 | 0 |
|  | 4-cell to 16-cell SG | 45 | 9 | 2 | 3 | 1 | 0 | 0 | 0 | 0 | 0 | 0 | 0 | 0 | 0 |
| Bam>R2 | GSC | 46 | 21 | 4 | 0 | 0 | 0 | 0 | 0 | 0 | 0 | 0 | 0 | 0 | 0 |
|  | GB & 2-cell SG | 50 | 12 | 7 | 1 | 1 | 0 | 0 | 0 | 0 | 0 | 0 | 0 | 0 | 0 |
|  | 4-cell to 16-cell SG | 22 | 14 | 12 | 11 | 7 | 1 | 3 | 0 | 0 | 0 | 0 | 0 | 0 | 1 |

**Table S1.** Testes count with each listed number of γH2Av positive cells in different germline cell types of control and Bam>R2 conditions (raw data for Figure 5C)
